# Actinoquinazolinone, a New Quinazolinone Derivative from a Marine Bacterium *Streptomyces* sp. CNQ-617, Suppresses the Motility of Gastric Cancer Cells

**DOI:** 10.3390/md21090489

**Published:** 2023-09-13

**Authors:** Sultan Pulat, Da-Ae Kim, Prima F. Hillman, Dong-Chan Oh, Hangun Kim, Sang-Jip Nam, William Fenical

**Affiliations:** 1 College of Pharmacy and Research Institute of Life and Pharmaceutical Sciences, Sunchon National University, Suncheon 57922, Republic of Korea; sultanpulat@s.scnu.ac.kr; 2 Department of Chemistry and Nanoscience, Ewha Womans University, Seoul 03760, Republic of Korea; 123rlaekdo@ewhain.net (D.-A.K.); primafitriah@gmail.com (P.F.H.); 3 Natural Products Research Institute, College of Pharmacy, Seoul National University, Seoul 08826, Republic of Korea; dongchanoh@snu.ac.kr; 4 Center of Marine Biotechnology and Biomedicine, Scripps Institution of Oceanography, University of California San Diego, La Jolla, CA 92093-0204, USA

**Keywords:** actinoquinazolinone, *Streptomyces* sp., marine natural products, quinazolinone, gastric cancer, motility

## Abstract

A HPLC-UV guided fractionation of the culture broth of *Streptomyces* sp. CNQ-617 has led to the isolation of a new quinazolinone derivative, actinoquinazolinone (**1**), as well as two known compounds, 7-hydroxy-6-methoxy-3,4-dihydroquinazolin-4-one (**2**) and 7-methoxy-8-hydroxy cycloanthranilylproline (**3**). The interpretation of 1D, 2D NMR, and MS spectroscopic data revealed the planar structure of **1**. Furthermore, compound **1** suppressed invasion ability by inhibiting epithelial–mesenchymal transition markers (EMT) in AGS cells at a concentration of 5 µM. In addition, compound **1** decreased the expression of seventeen genes related to human cell motility and slightly suppressed the signal transducer and activator of the transcription 3 (STAT3) signal pathway in AGS cells. Together, these results demonstrate that **1** is a potent inhibitor of gastric cancer cells.

## 1. Introduction

Cancer is the uncontrolled growth and division of cells in the body [1]. Gastric cancer, also known as stomach cancer, is responsible for an estimated 768,793 deaths, making it the fourth leading cause of cancer death in 2020 [2,3]. Suppressed metastasis may be a target in gastric cancer therapy [4]. During the epithelial—mesenchymal transition (EMT) process, cancer cells take on a mesenchymal cell phenotype to spread to a different part of the body. Therefore, EMT plays a significant role in metastatic cancers [5,6]. Furthermore, the signal transducer and activator of transcription 3 (STAT3) is a kind of oncogene that can promote the invasion and migration potential of cancer cells [7]. Thus, suppressing the EMT and STAT3 signal pathways is crucial for developing effective cancer therapies.

Marine microorganisms, including actinobacteria, have emerged as a promising source for the discovery of novel bioactive compounds with potential pharmacological properties [8,9]. Actinobacteria are Gram-positive bacteria that have been extensively studied for their ability to produce secondary metabolites with a wide range of biological activities. Many of these compounds have demonstrated promising activity against various diseases, including cancer, bacterial infections, and viral infections [10,11]. The high biodiversity of marine-derived actinobacteria provides a rich source of novel compounds, which is one of their main advantages [11]. Additionally, marine environments offer unique ecological niches, including extreme temperatures, pressures, and salinity, which can produce unique compounds not found in terrestrial environments. Thus, marine-derived actinobacteria are considered a valuable resource for drug discovery and development [10,11,12].

*Streptomyces* sp. strain CNQ-617 is a marine-derived actinobacterium isolated from sediment samples collected from La Jolla Submarine Canyon in California. This strain has been shown to produce several bioactive compounds with potential therapeutic applications [10]. *Streptomyces* is a well-known genus of actinobacteria known for producing a large number of secondary metabolites, including many clinically important antibiotics. Indeed, *Streptomyces* species are thought to have produced approximately two-thirds of all known antibiotics [11,13,14]. Researchers have also discovered several novel bioactive compounds derived from marine-derived *Streptomyces* species in recent years. 

Therefore, marine-derived *Streptomyces* and other actinobacteria are regarded as a valuable resource for the discovery of novel bioactive compounds with potential therapeutic applications [15,16,17,18,19,20,21,22,23,24,25,26]. Marineosins A and B are two structurally related compounds that were first discovered from this strain. They are analogs of the natural product prodigiosin and have been shown to possess strong and selective anticancer activity against a variety of cancer cell lines by inducing apoptosis, inhibiting angiogenesis, and disrupting microtubule assembly [26,27]. 

**Figure 1:**
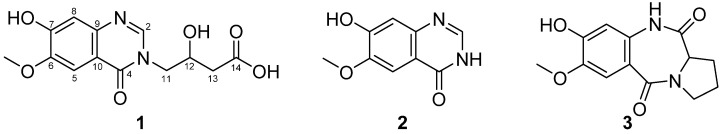
Chemical Structures of actinoquinazolione (**1**), 7-hydroxy-6-methoxy-3,4-dihydroquinazolin-4-one (**2**), and 7-methoxy-8-hydroxy cycloanthranilylproline (**3**).

In addition to marineosins A and B, this strain has also been shown to produce deoxyvasicinone, a compound with potential anti-melanogenic properties. Deoxyvasicinone has been shown to inhibit melanin production in murine and human melanoma cells, suggesting that it could be a promising agent for treating hyperpigmentation disorders [10]. Moreover, further extensive investigation of crude extracts from *Streptomyces* sp. strain CNQ-617 has resulted in the isolation of a novel quinazolinone derivative, actinoquinazolinone (**1**). This study reported the isolation of actinoquinazolinone (**1**) along with two known compounds, 7-hydroxy-6-methoxy-3,4-dihydroquinazolin-4-one (**2**) and 7-methoxy-8-hydroxy cycloanthranilylproline (**3**) (Figure 1), as well as the structural characterization and bioactivity of **1**.

## 2. Results and Discussion

Actinoquinazolinone (**1**) was isolated as a yellowish brown oil with a molecular formula of C_13_H_14_N_2_O_6_ based on the HR-FAB-MS ion at *m*/*z* 295.0924 [M + H]^+^ (calcd for C_13_H_15_N_2_O_6_, 295.0930). The ^1^H NMR spectrum of **1** (Table 1, Figures S1–S5) displayed one hetero-aromatic proton at *δ*_H_ 8.07 (^1^H, s, H-2), two aromatic protons at *δ*_H_ 7.46 (1H, s, H-5) and 6.98 (1H, s, H-8), one methoxy group at *δ*_H_ 3.88 (3H, s, 6-Ome), and one exchangeable proton at *δ*_H_ 10.35 (1H, s, OH). Based on spectroscopic data from ^13^C NMR and HSQC, six quaternary carbons were assigned at *δ*_C_ 159.7 (C-4), 148.2 (C-6), 153.0 (C-7), 143.9 (C-9), 113.8 (C-10), and 172.2 (C-14), two methylene carbons at *δ*_C_ 51.2 (C-11) and 39.8 (C-13), four methine carbons at *δ*_C_ 147.1 (C-2), 105.7 (C-5), 110.9 (C-8), and 65.0 (C-12), and one methoxy carbon at *δ*_C_ 55.7 (6-Ome).

**Figure 2:**
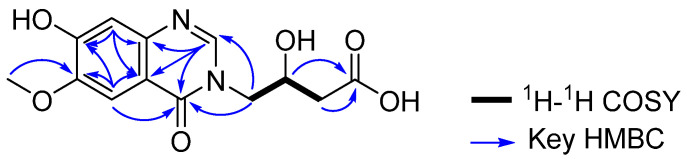
COSY and key HMBC correlations of **1**.

Additional 2D NMR analyses allowed the structure of **1** to be constructed. The quinazolin-4-one unit was assigned by the NMR signals of six aromatic carbons at *δ*_C_ 105.7 (C-5), 148.2 (C-6), 153.0 (C-7), 110.9 (C-8), 143.9 (C-9), and 113.8 (C-10), and two hetero-aromatic carbons at *δ*_C_ 147.1 (C-2) and 159.7 (C-4), along with the HMBC correlations from H-2 to C-4, C-9 and C-10; from H-5 to C-4, C-6, C-7 and C-10; and from H-8 to C-7, C-9 and C-10. In addition, HMBC correlations from 6-Ome to C-6 provided the attachment of the methoxy group at C-6. Furthermore, COSY cross peaks [H-11/H-12 and H-12/H-13] and long-range HMBC correlations of H-12 and H-13 with C-14 (*δ*_C_ 172.2) as well as the carbon chemical shift of C-12 (*δ*_C_ 65.0) allowed the construction of the 3-hydroxybutanoic acid moiety. This moiety was linked to the quinazolinone moiety through the nitrogen atom between C-2 and C-4 from the consideration of the carbon chemical shift of C-11 (*δ*_C_ 51.2) and from the observation of the long-range HMBC correlation from H-12 to C-2 and C-4. Additionally, the unassigned hydroxy group was determined to be located at C-7 based on the carbon chemical shift of C-7 (*δ*_C_ 153.0) and the molecular formula of the compound. Therefore, the final structure of actinoquinazolione (**1**) was determined to be 7-hydroxy-6-methoxy quinazolinone with a 3-hydroxy butanoic acid moiety, as shown in Figure 2.

The ^1^H NMR (400 MHz, DMSO-*d*_6_) spectrum of **2** revealed one hetero-aromatic proton at *δ*_H_ 7.90 (1H, d, *J* = 3.47, H-2), two aromatic protons at *δ*_H_ 7.43 (1H, s, H-5) and 6.98 (1H, s, H-8), one methoxyl group at *δ*_H_ 3.87 (3H, s, 6-Ome), and two exchangeable protons at *δ*_H_ 11.93 (1H, s, OH) and 10.27 (1H, br s, NH) (Figure S6). The interpretation of ^13^C NMR spectral data revealed nine carbons at *δ*_C_ 160.1 (C-4), 153.0 (C-7), 148.0 (C-2), 144.9 (C-6), 143.6 (C-9), 114.7 (C-10), 111.3 (C-8), 105.5 (C-5), and 55.7 (6-Ome) (Figure S7). Compound **2** was identified as 7-hydroxy-6-methoxy-3,4-dihydroquinazolin-4-one based on a comparison of its NMR data to that of a previously reported synthetic compound [28]. However, it was the first report of the compound being isolated from a natural source.

Compound (**3**) was isolated as yellowish oil and its molecular formula was determined to be C_13_H_14_N_2_O_4_ by HR-FAB-MS [M+H]^+^ ion at *m*/*z* 263.1029. The ^1^H NMR spectrum of compound **3** displayed *para*-coupled aromatic protons at *δ*_H_ 7.36 (1H, s, H-1) and 6.58 (1H, s, H-4) (Figure S8). The ^13^C NMR spectrum of **3** showed twelve carbons at *δ_C_* 171.2 (C-11), 166.6 (C-5), 151.1 (C-8), 145.4 (C-7), 131.4 (C-9a), 117.9 (C-5a), 111.8 (C-6), 107.7 (C-9), 57.2 (C-11a), 47.1 (C-3), 25.8 (C-1), 23.4 (C-2), and one methoxy group at *δ_C_* 55.4 (7-Ome) (Figure S9). The chemical structure of **3** was determined as 7-methoxy-8-hydroxy cycloanthranilylproline based on the comparison of NMR data to those of previously reported ones [25].

Compounds **1** and **2** are quinazolinone derivatives with an *N*-containing heterocyclic scaffold. Quinazolinones are a class of nitrogen-containing heterocyclic compounds found in nature which are produced by plants and microorganisms [29,30]. Quinazolinones have sparked considerable interest in the fields of medicinal chemistry and drug discovery. The chemical structure of quinazolinones has been shown to possess a wide range of pharmacological properties [31]. Furthermore, quinazolinones are considered as a significant scaffold of various therapeutic and biological activities, including anticancer [32], anticonvulsant [33], anti-cholinesterase [34], anti-diabetic [35], antimalarial [36], antimicrobial [37,38], antitubercular [39], antihypertensive [40], anti-HIV [41], anti-inflammatory [42], and antipsychotic [43]. Other therapeutic and biological activities include cellular phosphorylation inhibition [44], kinase inhibitory [45], dihydrofolate reductase inhibition [46], inhibitors of tubuline polymerization [47], dopamine agonists, and diuretic activities [48,49]. Reported microbial-derived quinazolinones, penicamide A, penoxazolones A and B, aspertoryadins A–J, nortryptoquivaline, 2-(4-hydroxybenzyl)quinazolin-4(3H)-one, and penipanoids B and C, are isolated from the ascidian-derived fungus *Penicillium* sp. 4829 [50], the cold-seep-derived fungus *Penicillium oxalicum* [51], the marine-derived fungus *Aspergillus* sp. HNMF114 [52,53], the sea fan-derived fungus *Neosartorya siamensis* [54], the entomopathogenic fungus *Isaria farinose* [55], and the sediment-derived fungus *Penicillium paneum* SD-44 [56], respectively. In addition, several quinazolinones from *Streptomyces* have been reported, including 2-(1H-indol-3-yl)quinazolin-4-(3H)-one, quinazolin-4(3H)-one, 2-methylquinazolin-4(3H)-one [57], 2-(4-hydroxyphenyl) quinazolin-4(3H)-one [50], quinazolinones A and B, 4(3H)-quinazolinone [58], 2-(2-carboxyethyl)-8-hydroxyquinazolin-4(3H)-one, 2-(2-carboxyethyl)-6-hydroxyquinazolin-4(3H)-one, 2-(4-hydroxyphenyl)quinazolin-4(3H)-one [59], farinamycin [60], 2-Methyl-3H-quinazolin-4-one and 1H-quinazoline-2,4-dione and arborine [61,62]. Moreover, these quinazolinones have been reported to have various biological activities, such as cytotoxicity to Vero cells [63], antifungal activity against *P. litchi* [64], and cytotoxicity against KB and HL-60 cell lines [65].

**Figure 3:**
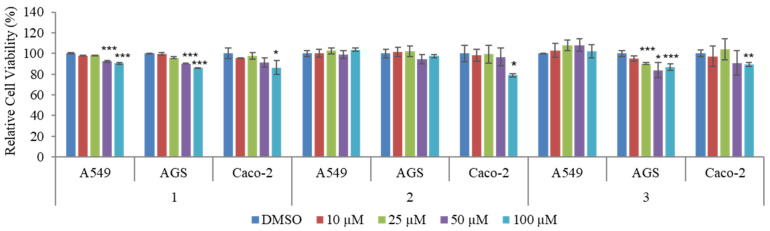
The effect of different concentrations of actinoquinazolione (**1**), 7-hydroxy-6-methoxy-3,4-dihydroquinazolin-4-one (**2**), and 7-methoxy-8-hydroxy cycloanthranilylproline (**3**) on cell viability of AGS, A549, and Caco-2. Cell viability was measured by using MTT assays. Data are presented as mean ± SD and analysis was performed by Student’s *t*-test (*n* = 3). * *p* < 0.05; ** *p* < 0.01; *** *p* < 0.001.

Additionally, compounds **1** and **2** share structural similarities with previously reported compound 4-(7,8-dihydroxy-4-oxoquinazolin-3(4H)-yl) butanoic acid, which was isolated from the leather coral-derived fungus *Xylaria* sp. FM1005 [66]. However, the structures of compounds **1** and **2** differ from that of the previously reported compound. In the quinazolinone moiety, compound **1** has a substituted methoxy group at C-6 and an additional hydroxy group attached at C-12, but no 8-OH group. Meanwhile, compound **2** has a similar structure to compound **1**, except for the absence of the 3-hydroxybutanoic acid substituent on the amide moiety. In addition, **1**–**3** have been tested for their viability of cancer cell lines. The relative cell viability of cancer cell lines A549 (lung cancer), AGS (gastric cancer), and Caco-2 (colorectal cancer) was measured by an MTT assay after treatment with various concentrations of **1**–**3** for 48 h. The result showed that treatment with 100 μM of compound **1** significantly decreased the cell viability of A549, AGS, and Caco-2. However, 100 μM of compound **2** did not affect the cell viability of A549 and AGS, while suppressing the cell viability of Caco-2. Moreover, 100 μM of compound **3** suppressed the cell viability of AGS and Caco-2 while having no significant effect on the cell viability of A549 (Figure 3). Therefore, these results showed that compound **1** is more effective in reducing the cell viability of A549, AGS, and Caco-2 than the other two compounds. However, it should be noted that the observed cell viability reduction by **1**–**3** is modest even at 100 μM.

**Figure 4:**
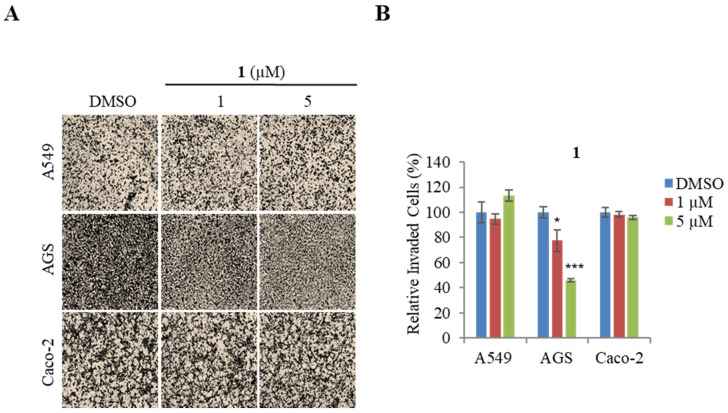
Effect of actinoquinazolione (**1**) on the invasion ability of AGS, A549, and Caco2 cells. (**A**) representative images of each insertion in the invasion assay. (**B**) relative percentage of invaded cells. Data are presented as the mean ± SD and analysis was performed by Student’s *t*-test (*n* = 5). * *p* < 0.05, *** *p* < 0.001.

To confirm the concentration-dependent inhibitory effect of compound **1** on cancer motility, we evaluated invasion assays in A549, AGS, and Caco-2 cells. From the results (Figure 4A,B), **1** displayed a dose-dependent inhibitory effect on the invasion ability of AGS at concentrations from 1 to 5 μM, whereas the invasion ability of A549 and Caco-2 cells were not affected by treatment of **1**. The results indicated that **1** has a higher suppression activity on the AGS invasion ability than A549 and Caco-2.

**Figure 5:**
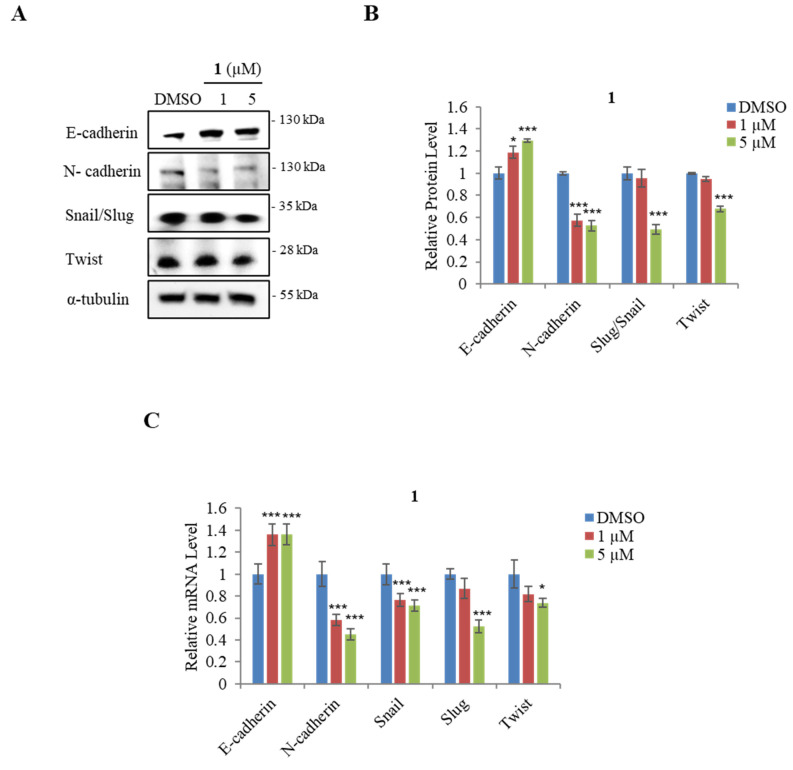
Effect of actinoquinazolione (**1**) on EMT markers in AGS cells. (**A**) Western blot analysis of E-cadherin, N-cadherin Snail/Slug, and Twist. (**B**) relative protein levels of E-cadherin, N-cadherin Snail/Slug, and Twist. (**C**) relative mRNA expression levels of E-cadherin, N-cadherin Snail, Slug, and Twist. mRNA expression levels were normalized against the glyceraldehyde 3-phosphate dehydrogenase (GAPDH) housekeeping gene. Data are presented as mean ± SD and analysis was performed by Student’s *t*-test (*n* = 3).* *p* < 0.05; *** *p* < 0.001.

Cancer metastasis accounts for approximately 90% of cancer deaths [67,68]. A poor prognosis can be caused by the EMT, which is linked to metastasis [69]. E-cadherin is known to suppress cancer metastasis; the loss of its expression promotes the EMT markers E-cadherin, Snail, Slug, and Twist [69]. We assessed whether compound **1** decreased the motility associated with EMT by using qPCR assays and Western blot assays (Figure 5A). Based on the result, treatment with 5 μM of **1** significantly promoted the protein and mRNA expression level of E-cadherin in AGS cells (Figure 5B,C). Furthermore, the protein and mRNA expression levels of the EMT effector N-cadherin and the EMT transcription factors Snail, Slug, and Twist were decreased in AGS by treatment with 5 μM of **1** (Figure 5B,C).

**Figure 6:**
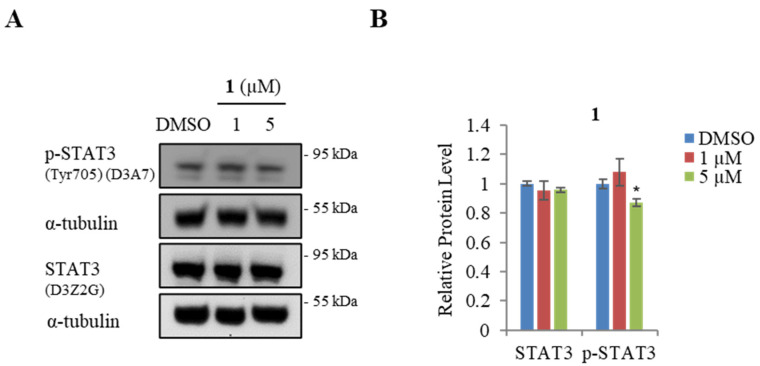
Effect of actinoquinazolione (**1**) on STAT3 in AGS cells. (**A**) Western blot analysis of STAT3 and p-STAT3. (**B**) relative protein levels of STAT3 and p-STAT3. Data are presented as mean ± SD and analysis was performed by Student’s *t*-test (*n* = 3). * *p* < 0.05.

Previous studies have shown that STAT3 and EMT interact with each other to promote cancer metastasis. EMT is the downstream mediator of STAT3 and, therefore, the upregulation of the positive effect of STAT3 on the EMT process [70]. Western blot assays were performed to assess whether treatment with **1** affects the STAT3 protein level. The results (Figure 6A,B) indicate that **1** did not affect the total STAT3 protein level, whereas 5 μM of **1** slightly decreased the protein level of phosphorylated STAT3 (p- STAT3) in AGS cells.

**Figure 7:**
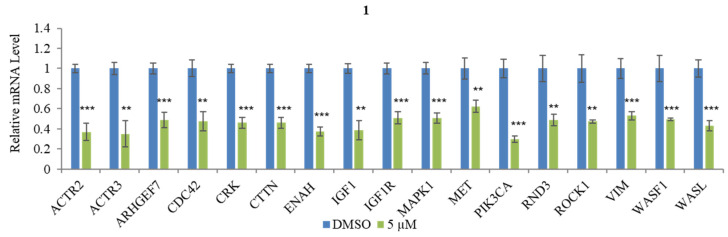
Effect of actinoquinazolione (**1**) on cell motility-related genes in AGS cells Relative mRNA expression levels of ACTR2/3, ARHGEF7, CDC42, CRK, CTTN, ENAH, IGF-1, IGF1R, MAPK1, MET, PIK3CA, RND3, ROCK1, VIM, WASF1, and WASL in AGS cells treated with 5μM of **1**. Data are presented as mean ± SD and analysis was performed by Student’s *t*-test (*n* = 3). ** *p* < 0.01; *** *p* < 0.001.

Finally, the Human Cell Motility RT2 Profiler PCR Array was used to examine the effect of compound **1** on the mRNA expression level of cell motility-related genes. The results (Figure 7) showed that treatment with 5 μM of **1** decreased the expression of ARP2/3 actin-related protein 2/3 (ACTR2/3), rho guanine nucleotide exchange factor (GEF) 7 (ARHGEF7), cell division cycle 42 (CDC42), V-crk sarcoma virus CT10 oncogene homolog (CRK), cortactin (CTTN), enabled homolog (ENAH), insulin-like growth factor 1 (IGF-1), insulin-like growth factor 1 receptor (IGF1R), mitogen-activated protein kinase 1 (MAPK1), Met proto-oncogene (hepatocyte growth factor receptor (MET), Phosphoinositide-3-kinase, catalytic (PIK3CA), rho family GTPase 3 (RND3), rho-associated, coiled-coil containing protein kinase 1 (ROCK1), Vimentin (VIM), WAS protein family 1 (WASF1), and walkout-Aldrich syndrome protein-like (WASL). These genes are related to cancer cell motility and could be used to treat cancer. ACTR2/3 regulates cell motility by playing an important role in actin dynamics and cytoskeleton organization [71]. The ARHGEF7 gene stimulates cancer cell motility and invasiveness by modifying the cytoskeleton [72]. CDC42 is as known a structural homolog of the Rho GTPase family; inhibition of CDC42 can reduce cancer progression by suppressing distinct GEFs [73]. CRK is a regulator of kinase and overexpression of CRK is associated with adenocarcinomas of the stomach [74]. CTTN is a regulator of actin polymerization by binding the actin-regulated protein complex ACTR2/3 [75]. ENAH includes a member of the ENAH/VASP family which can modify cell morphology and adhesion in the metastasis process [76]. IGF1 promotes cell proliferation by suppressing apoptosis in cancer [77]. MAPK1 is closely related to invasion and metastasis via modulated EMT [78]. The invasiveness and metastasis of aggressive cancer cells link to the overexpression of oncogene MET [79]. PIK3CA has been related to cancer cell motility, which is the second most frequent mutant oncogene. A statistical analysis showed that mutation of PIK3CA is the reason for more than 10% of cancer cases [80]. RND3 may serve as a predictor of EMT upregulation [81]. ROCK1 is a small GTPase Rho downstream effector that is crucial in cancer metastasis [82]. VIM has been related to cancer metastasis by promoting the EMT process [83]. WASF1, also known as WAVE1 (WASP family verprolin homologous protein 1), is associated with regulating actin cytoskeleton dynamics for cancer cell invasion and migration [84].

## 3. Materials and Methods

### 3.1. General Experimental Procedures

Optical rotations were measured using a Kruss Optronic P-8000 polarimeter with a 5 cm cell. Infrared spectra were measured with a Varian Scimitar Series FT-IR spectrometer in methanol (MeOH). The NMR spectra were established by an Agilent NMR spectrometer (Agilent, Santa Clara, CA, USA, at 400 MHz for ^1^H and at 100 MHz for ^13^C) equipped at the Drug Development Research Core Center using the signals of the residual solvent as internal references (*δ*_H_ 2.50 ppm and *δ*_C_ 39.5 ppm for dimethyl sulfoxide-*d*_6_ (DMSO-*d*_6_) and *δ*_H_ 3.31 ppm and *δ*_C_ 4.91 ppm for deuterated methanol (CD_3_OD). The low-resolution LC/MS measurements were recorded on the Agilent Technologies 1260 quadrupole and Waters Micromass ZQ LC/MS system using a reversed-phase column (Phenomenex Luna C18 (2) 100 Å, 50 mm × 4.6 mm, 5 µm) at a flow rate of 1.0 mL/min at the National Research Facilities and Equipment Center (NanoBioEnergy Materials Center) at Ewha Womans University. Open column chromatography was performed using silica (40–63 µm, Merck silica gel 60, Kenilworth, NJ, USA) eluting with a gradient solvent of dichloromethane (CH_2_Cl_2_) and methanol (MeOH). The fractions were purified via semi-preparative HPLC using a Waters 996 Photodiode Array Detector HPLC coupled with a reversed-phase Phenomenex Luna C18 (2) (100 Å, 250 nm × 10 mm, 5 µm) column with a mixture of acetonitrile and H_2_O at a flow rate of 2.0 mL/min. High-resolution mass spectra were recorded on a JMS-700 mass spectrometer (JEOL Ltd., Tokyo, Japan) at Seoul National University.

### 3.2. Collection and Phylogenetic Analysis of the CNQ-617 Strain

The marine actinomycete strain CNQ-617 was isolated from a marine sediment sample collected offshore of La Jolla, California. The strain was specified as the MAR3 clade based on 16S rDNA analysis. The phylogenetic analysis revealed that this strain showed 99.7% similarity to *Streptomyces cacaoi* based upon the result of NCBI blast analysis of the partial 16S rDNA. The gene sequence data are available from GenBank (deposit #EU161093).

### 3.3. Cultivation and Extraction

*Streptomyces* strain CNQ-617 was cultured in 160 of 2.5-L Ultra Yield Flasks each containing 1 L of the medium (10 g/L of soluble starch, 2 g/L of yeast, 4 g/L of peptone, 10 g/L of CaCO_3_, 20 g/L of KBr, 8 g/L Fe_2_(SO_4_)_3_·4H_2_O dissolved in 750 mL of natural seawater and 250 mL of distilled water) at 27 °C with constant shaking at 120 rpm. After 15 days, the culture broth was extracted with ethyl acetate (EtOAc; 160 L in total) to obtain 16.0 g of EtOAc extract. 

### 3.4. Isolation of Compounds

The crude extract (16.0 g) of the CNQ-617 strain was fractionated by medium-pressure liquid chromatography (MPLC) eluting with a step gradient of CH_2_Cl_2_ and MeOH (100/0, 99/1, 98/2, 96/4, 95/5, 90/10, 80/20, 50/50, 0/100, *v/v*, 600 mL for each gradient) to afford fractions M1–M9. The fourth fraction M4 was purified by HPLC (Phenomenex Luna C18(2) 100 Å, 250 mm × 10 mm, 5 µm) with 15% acetonitrile in H_2_O with 0.1% trifluoroacetic acid (TFA) at a flow rate of 2.0 mL/min to yield 19.0 mg actinoquinazolinone (**1**, t_R_ 14.5 min) and 7-methoxy-8-hydroxy cycloanthranilylproline (**3**, t_R_ 27.0 min). Fraction M8 was also purified by reversed phase HPLC (Phenomenex 100 Å, 250 mm × 10 mm, 5 µm,) under isocratic conditions with 11% acetonitrile in H_2_O with 0.1% TFA at flow rate 2.0 mL/min to yield 7-hydroxy-6-methoxy-3,4-dihydroquinazolin-4-one (**2**, t_R_ 15.9 min).

*Actinoquinazolinone* (**1**): yellowish brown oil; ${\left[\alpha \right]}_{D}^{25}$ = +62.4 (*c* 0.19, MeOH); UV (MeOH) λ_max_ (log ε) 202 (2.4), 243 (2.7), 309 (1.9), 322 (1.9) nm; IR (KBr) ν_max_ 3188, 2956, 2925, 1658, 1457 cm^−1 (^Figure S10), ^1^H and ^13^C NMR data, Table 1, Figures S1–S5; HR-FAB-MS *m/z* 295.0924 [M + H]^+^ (Figure S11, calcd for C_13_H_15_N_2_O_6_, 296.0930). 

*7-Hydroxy-6-methoxy-3,4-dihydroquinazolin-4-one* (**2**): ^1^H (400 MHz, DMSO-*d_6_*, Figure S6); *δ*_H_ 7.91 (s, H-2), 7.43 (s, H-5), 6.98 (s, H-8), 3.87 (s, 6-OMe), ^13^C (100 MHz, DMSO-*d_6_*, Figure S7); *δ_C_* 160.1 (C-4), 153.0 (C-7), 148.0 (C-2), 144.9 (C-6), 143.6 (C-9), 114.7 (C-10), 111.3 (C-8), 105.5 (C-5), 55.7 (6-OMe), LR-ESI-MS *m/z* 193.1 [M + H]^+^.

*7-methoxy-8-hydroxy cycloanthranilylproline* (**3**): ${\left[\alpha \right]}_{D}^{25}$ = +275 (c 0.22, MeOH); IR (KBr) v_max_ 3214, 1693, 1607, 1519, 1437, 1274, 1201, 1179, 786 cm^−1^; UV (MeOH) λ_max_ (log ε) 220 (7.76), 260 (3.79), 300 (3.31) nm, ^1^H (500 MHz, CD_3_OD, Figure S8); *δ*_H_ 7.36 (s, H-6), 6.58 (s, H-9), 4.16 (m. H-11a), 3.91 (s, 7-OCH_3_), 3.72 (m, H-3), 3.57 (m, H-3), 2.67 (m, H-1), 2.05 (m, H-1), 2.02 (m, H-2), ^13^C (125 MHz, CD_3_OD, Figure S9); *δ_C_* 171.2 (C-11), 166.6 (C-5), 151.1 (C-8), 145.4 (C-7), 131.4 (C-9a), 117.9 (C-5a), 111.8 (C-6), 107.7 (C-9), 57.2 (C-11a), 55.4 (7-OCH_3_), 47.1 (C-3), 25.8 (C-1), 23.4 (C-2), HR-FAB-MS *m/z* 263.1029 [M + H]^+^ (calcd for C_13_H_14_N_2_O_4_, 263.1029).

### 3.5. Cell Culture 

Cell lines A549 (lung cancer), AGS (gastric cancer), and Caco-2 (colorectal cancer) were cultured in Roswell Park Memorial Institute (RPMI) 1640 Medium or Dulbecco’s Modified Eagle Medium (DMEM) (Gen Depot, Barker, TX, USA), supplemented with 10% FBS and 1% penicillin–streptomycin solution C in a humid environment with 5% CO_2_ [85].

### 3.6. MTT Assay 

A549 (3 × 10^3^ cells/well), AGS (3 × 10^3^ cells/well), and Caco-2 (2.5 × 10^3^ cells/well) were seeded on 96-well plates for gown overnight, and then treated with DMSO (Sigma-Aldrich) or various concentrations of actinoquinazolione (**1**), 7-hydroxy-6-methoxy-3,4-dihydroquinazolin-4-one (**2**), and 7-methoxy-8-hydroxy cycloanthranilylproline (**3**) for 48 h. MTT was added to the cultures for 4 h after treatment. In the final step of MTT, the cells were lysed with 150 µL of DMSO and the absorbance was measured using a spectrophotometer (Bio Tek Instruments, Winooski, VT, USA) [86,87].

### 3.7. Invasion Assay 

Transwell containing polycarbonate membranes with 8 µm pores coated with 1% gelatin-coated polycarbonate was used to measure the invasion ability of AGS cells. The AGS cells were seeded in media containing 0.2% bovine serum albumin (BSA), then treated with 1 and 5 µM concentrations of compound **1** for 24 h and DMSO as a control. The lower chamber was filled with 600 µL RPMI containing 0.2% BSA and 6 µg/mL fibronectin (EMD Millipore Corp., Billerica, MA, USA) as a chemoattractant. Diff-Quik kit (Sysmex, Kobe, Japan) was used for the fixation and dying of AGS cells after 24 h treatment, the cells in the upper chambers were quantified using a *K1*-*Fluo* Confocal *Microscope* (Nanoscope Systems, Republic of Korea) and i-Solution FL Auto Software (IMT i-Solution Inc., Vancouver, QC, Canada [88,89].

### 3.8. qPCR 

Total RNA of AGS cells was extracted by RNAiso Plus (Takara, Otsu, Japan) according to the manufacturer’s suggestions. Using a Moloney Murine Leukemia Virus (M-MLV) kit, total RNA (1 µg) from DMSO as well as 1 and 5 μM of treatment with compound **1** groups were converted to cDNA. SYBR Green (Enzynomics, Seoul, Republic of Korea) was used to evaluate relative gene expression and CFX (Bio-Rad, Hercules, CA, USA) was applied for analysis [90,91].

### 3.9. Western Blots 

The AGS cells were seeded in 6-well plates for gown overnight, and then treated with DMSO as well as 1 and 5 µM concentrations of **1**. After 24 h treatment, rinsed with PBS, then extracted using lysis buffer. The extracted protein was separated by SDS-PAGE. The membranes were incubated in blocking buffer (20 mmol/l Tris-HCl, 137 mmol/l NaCl, pH 7.6, 492 containing 0.1% Tween and 3% nonfat dry milk) for one hour and antibodies against E-cadherin and N-cadherin (BD Bioscience, San Jose, CA, USA); Snail/Slug and Twist (Abcam, London, UK); α-tubulin, STAT3, and p-STAT3 (Cell Signaling Technology, Danvers, MA, USA) were detected by horseradish peroxidase-conjugated secondary antibodies (Thermo Fisher Scientific, Waltham, MA, USA) using the Immobilon Western Chemiluminescent HRP Substrate Kit (Millipore, Billerica, MA, USA). The Multi Gauge 3.0 (Fujifilm, Tokyo, Japan) software was used for analyzing the density of the bands [92,93]. 

### 3.10. Statistical Analysis 

Data is represented as means ± standard deviation. All statistical analyses were carried out using the Sigma Plot software. Student’s *t*-test was used to compare statistically significant differences between two groups. A *p*-value less than 0.05 was considered statistically significant. 

## 4. Conclusions

In summary, the exploration of marine natural products from marine sediment-derived *Streptomyces* sp. CNQ-617 has led to the discovery of a novel compound, actinoquinazolinone (**1**), along with two previously reported compounds, 7-hydroxy-6-methoxy-3,4-dihydroquinazolin-4-one (**2**) and 7-methoxy-8-hydroxy cycloanthranilylproline (**3**). Furthermore, compound **1** exhibited moderate antibacterial activity against the Gram-positive bacteria *K*. *rhizophila* KCTC 1915 and weak inhibitory activities against *B. subtilis* KCTC 1021 and *S. aureus* KCTC 1927. In addition, compound **1** showed higher activity than compounds **2** and **3** in decreasing the cell viability of cancer cells. Moreover, compound **1** suppressed the invasion ability of the EMT and STAT3 signal pathways, and some of the cell motility-related genes. These findings highlight the potential of marine sediment-derived *Streptomyces* sp. CNQ-617 as a source of novel bioactive compounds that may have therapeutic applications. Further studies are required to determine the potential therapeutic effect and the potential side effect of **1**.

## Figures and Tables

**Table 1:** NMR Spectral Data for Compounds **1** (DMSO-*d*_6_) ^a.^

No.	1			
	*d_C_*, mult. ^b^	*d_H_* (*J* in Hz)	COSY	HMBC
2	147.1, CH	8.07, s		4, 9, 10, 11
4	159.7, qC			
5	105.7, CH	7.46, s		4, 6, 7, 8, 9, 10
6	148.2, qC			
7	153.0, qC			
8	110.9, CH	6.98, s		4, 6, 7, 9, 10
9	143.9, qC			
10	113.8, qC			
11a	51.2, CH_2_	3.74, dd (13.4, 8.2)	12	2, 4, 12, 13
11b		4.12, dd (13.4, 3.9)		
12	65.0, CH	4.18, m	13	
13a	39.8, CH_2_	2.29, dd (15.5, 8.2)		11, 12, 14
13b		2.45, dd (15.5, 4.7)		
14	172.2, qC			
6-Ome	55.7, CH_3_	3.88, s		6
7-OH		10.35, s		
12-OH				
14-OH				
14-Ome				

^a^ 400 MHz for ^1^H NMR and 100 MHz for ^13^C NMR. ^b^ Numbers of attached protons were determined by analysis of 2D spectra.

## Data Availability

The data presented in this study are available on request from the corresponding author.

## Supplementary Materials

The following are available online at https://www.mdpi.com/article/10.3390/md21090489/s1, Figures S1–S5 NMR spectra of actinoquinazolinone (**1**) in DMSO-d*_6_*; Figures S6 and S7 NMR spectra of 7-hydroxy-6-methoxy-3,4-dihydroquinazolin-4-one (**2**) in DMSO-d*_6_*; Figures S8 and S9 NMR spectra of 7-methoxy-8-hydroxy cycloanthranilylproline (**3**) in CD_3_OD; Figures S10 and S11 IR and HRMS spectra actinoquinazolinone (**1**).
