# Supplementary material for: Actinoquinazolinone, a New Quinazolinone Derivative from a Marine Bacterium Streptomyces sp. CNQ-617, Suppresses the Motility of Gastric Cancer Cells
Source: Mar Drugs. 2023 Sep 13;21(9):489. doi: 10.3390/md21090489 (PMC10532864; doi:10.3390/md21090489)
Supplement: Supplementary file 1 [file marinedrugs-21-00489-s001.zip › marinedrugs-2587655-supplementary.pdf]

Supplementary Material

Actinoquinazolinone, a New Quinazolinone Derivative from a Marine Bacterium *Streptomyces* sp. CNQ-617, Suppresses the Motility of Gastric Cancer Cells

Sultan Pulat [1,†], Da-Ae Kim [2,†], Prima F. Hillman [2,†], Dong-Chan Oh [3], Hangun Kim [1,*], Sang-Jip Nam [2,*] and William Fenical [4,*]

[1] College of Pharmacy and Research Institute of Life and Pharmaceutical Sciences, Sunchon National University, Sunchon 57922, Republic of Korea; sultanpulat@s.scnu.ac.kr

[2] Department of Chemistry and Nanoscience, Ewha Womans University, Seoul 03760, Republic of Korea; 123rlaekdo@ewhain.net (D.-A.K.); primafitriah@gmail.com (P.F.H.)

[3] Natural Products Research Institute, College of Pharmacy, Seoul National University, Seoul 08826, Republic of Korea; dongchanoh@snu.ac.kr

[4] Center of Marine Biotechnology and Biomedicine, Scripps Institution of Oceanography, University of California San Diego, La Jolla, CA 92093-0204, USA

* Correspondence: hangunkim@sunchon.ac.kr (H.K.); sjnam@ewha.ac.kr (S.-J.N.); wfenical@ucsd.edu (W.F.)

† These authors contributed equally to this work.

Table of Contents

Figure S1. ^{1}H NMR Spectrum (400 MHz) of actinoquinazolinone (**1**) in DMSO-d_6 3

Figure S2. ^{13}C NMR Spectrum (100 MHz) of actinoquinazolinone (**1**) in DMSO-d_6 4

Figure S3. COSY Spectrum (400 MHz) of actinoquinazolinone (**1**) in DMSO-d_6 5

Figure S4. HSQC Spectrum (400 MHz) of actinoquinazolinone (**1**) in DMSO-d_6 6

Figure S5. HMBC Spectrum (400 MHz) of actinoquinazolinone (**1**) in DMSO-d_6 7

Figure S6. ^{1}H NMR Spectrum (400 MHz) of 7-hydroxy-6-methoxy-3,4-dihydroquinazolin-4-one (**2**) in DMSO-d_6 8

Figure S7. ^{13}C NMR Spectrum (100 MHz) of 7-hydroxy-6-methoxy-3,4-dihydroquinazolin-4-one (**2**) in DMSO-d_6 9

Figure S8. ^{1}H NMR Spectrum (500 MHz) of 7-methoxy-8-hydroxy cycloanthranilylproline (**3**) in CD_3OD..................... 10

Figure S9. ^{13}C NMR Spectrum (125 MHz) of 7-methoxy-8-hydroxy cycloanthranilylproline (**3**) in CD_3OD.................... 11

Figure S10. IR Spectrum of actinoquinazolinone (**1**) 12

Figure S11. HRMS Spectrum of actinoquinazolinone (**1**) 13

Figure S1. ^{1}H NMR Spectrum (400 MHz) of actinoquinazolinone (**1**) in DMSO-d_6

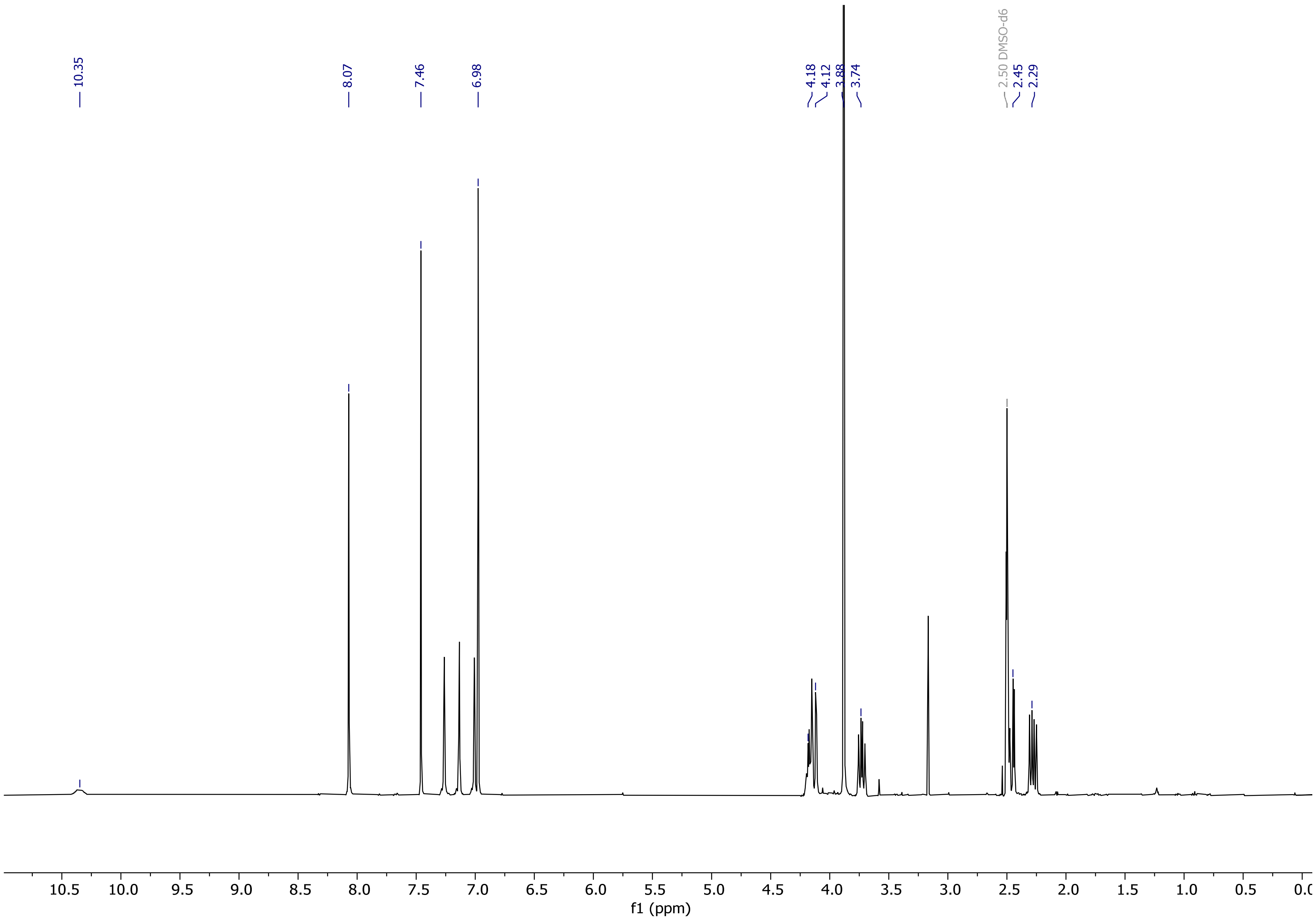

Figure S2. ^{13}C NMR Spectrum (100 MHz) of actinoquinazolinone (**1**) in DMSO-d_6

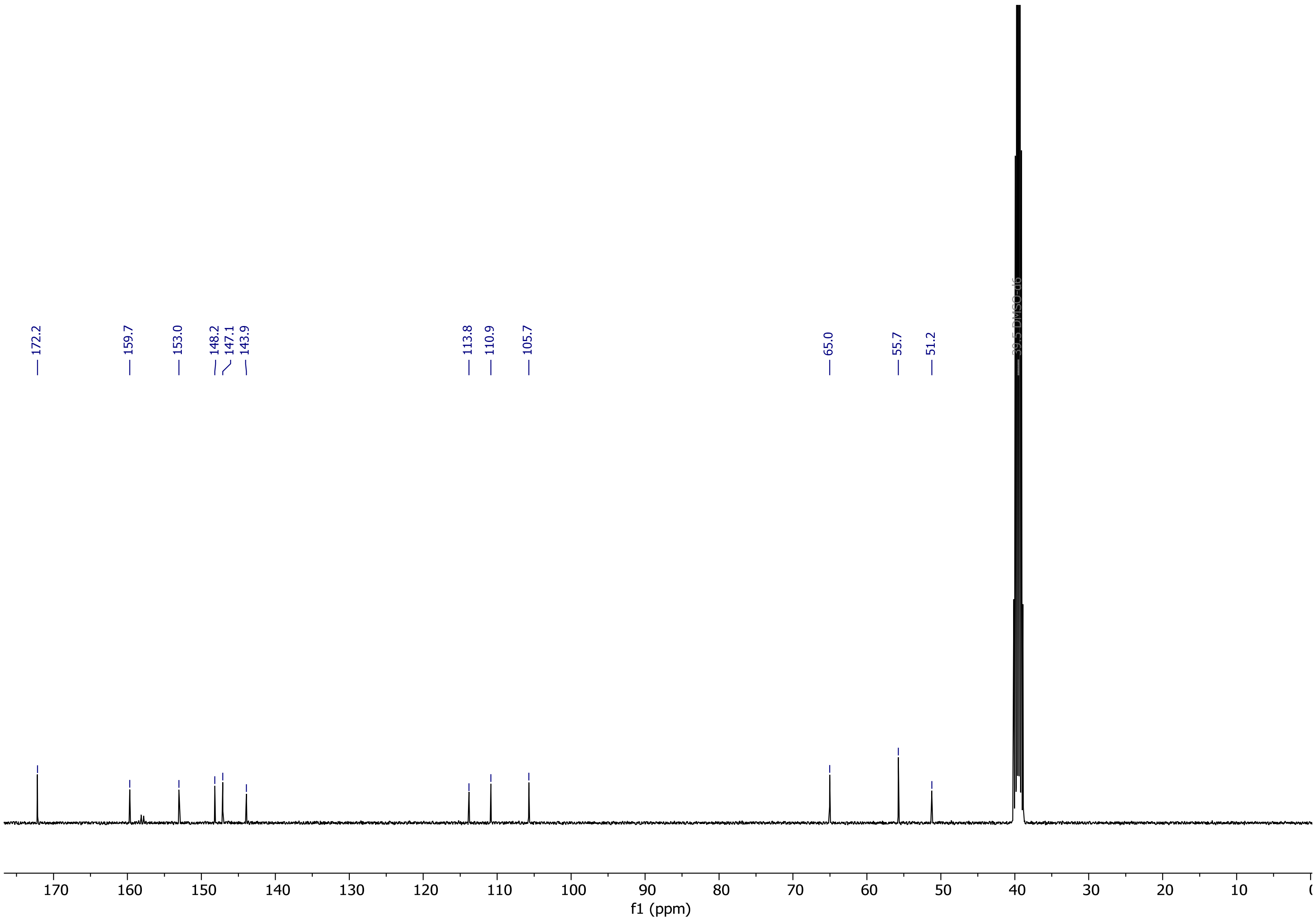

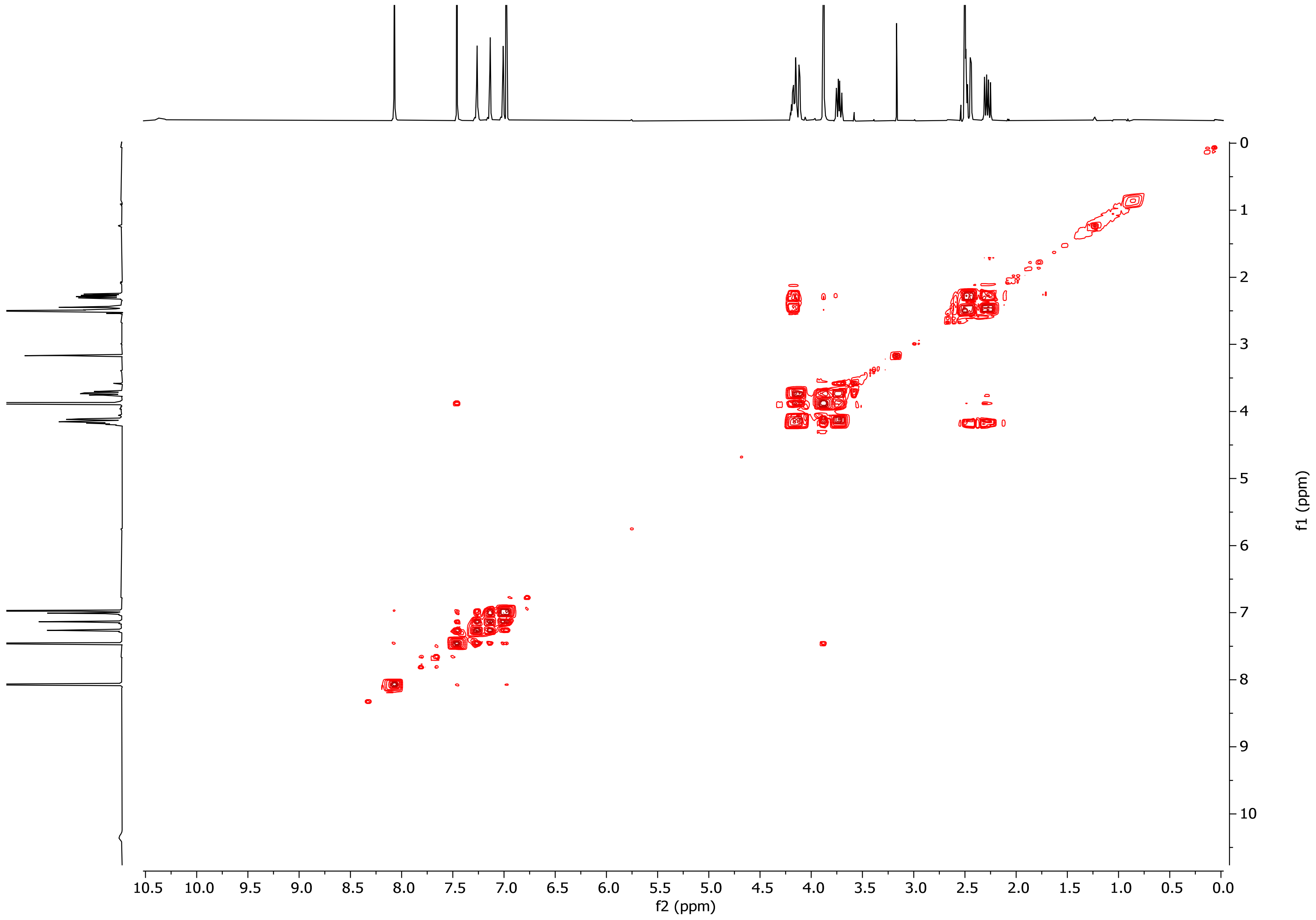

Figure S3. COSY Spectrum (400 MHz) of actinoquinazolinone (**1**) in DMSO-d_6

Figure S4. HSQC Spectrum (400 MHz) of actinoquinazolinone (**1**) in DMSO-d_6

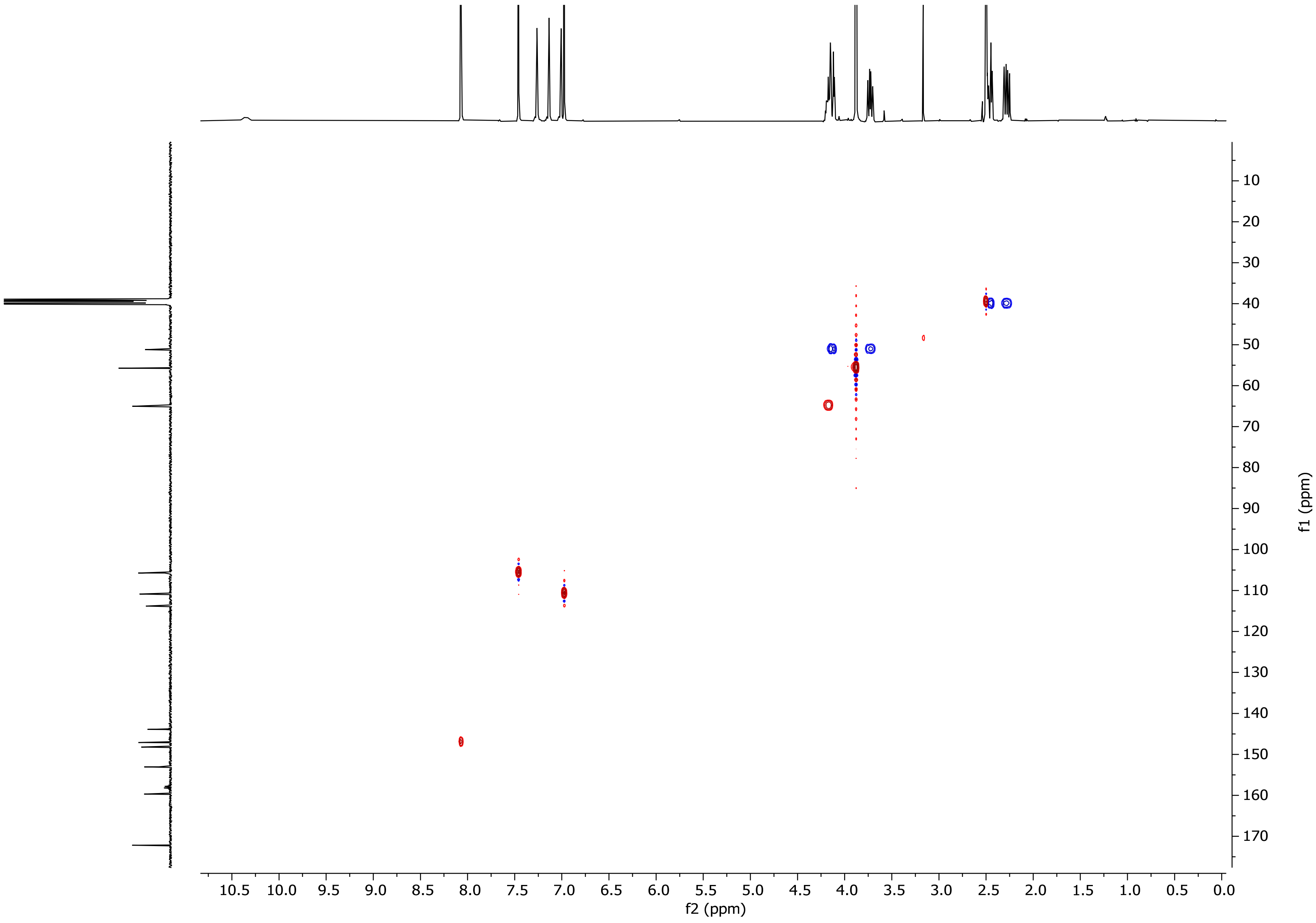

Figure S5. HMBC Spectrum (400 MHz) of actinoquinazolinone (**1**) in DMSO-*d*₆

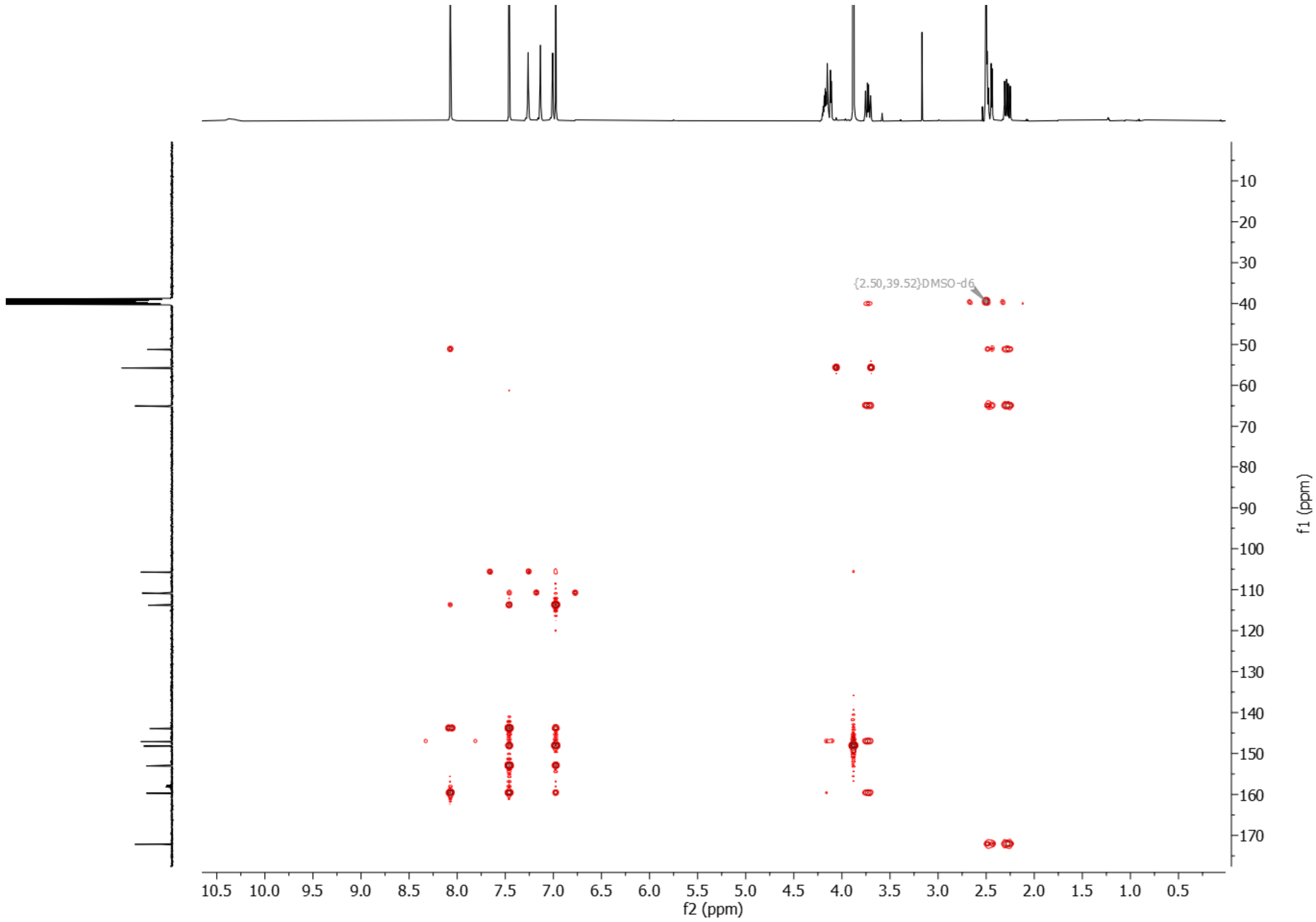

Figure S6. ^{1}H NMR Spectrum (400 MHz) of 7-hydroxy-6-methoxy-3,4-dihydroquinazolin-4-one (**2**) in DMSO-d_6

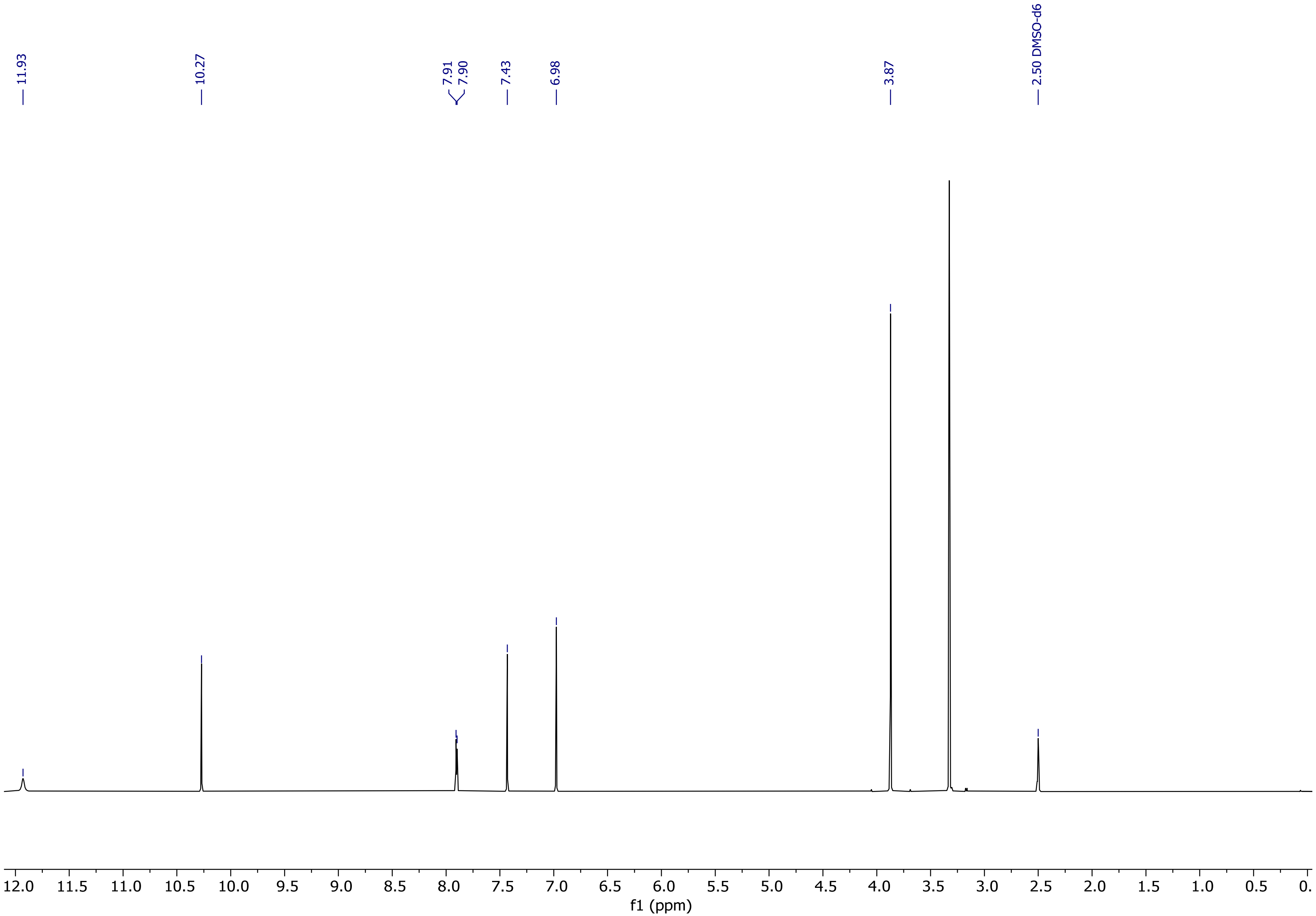

Figure S7. ^{13}C NMR Spectrum (100 MHz) of 7-hydroxy-6-methoxy-3,4-dihydroquinazolin-4-one (**2**) in DMSO-d_6

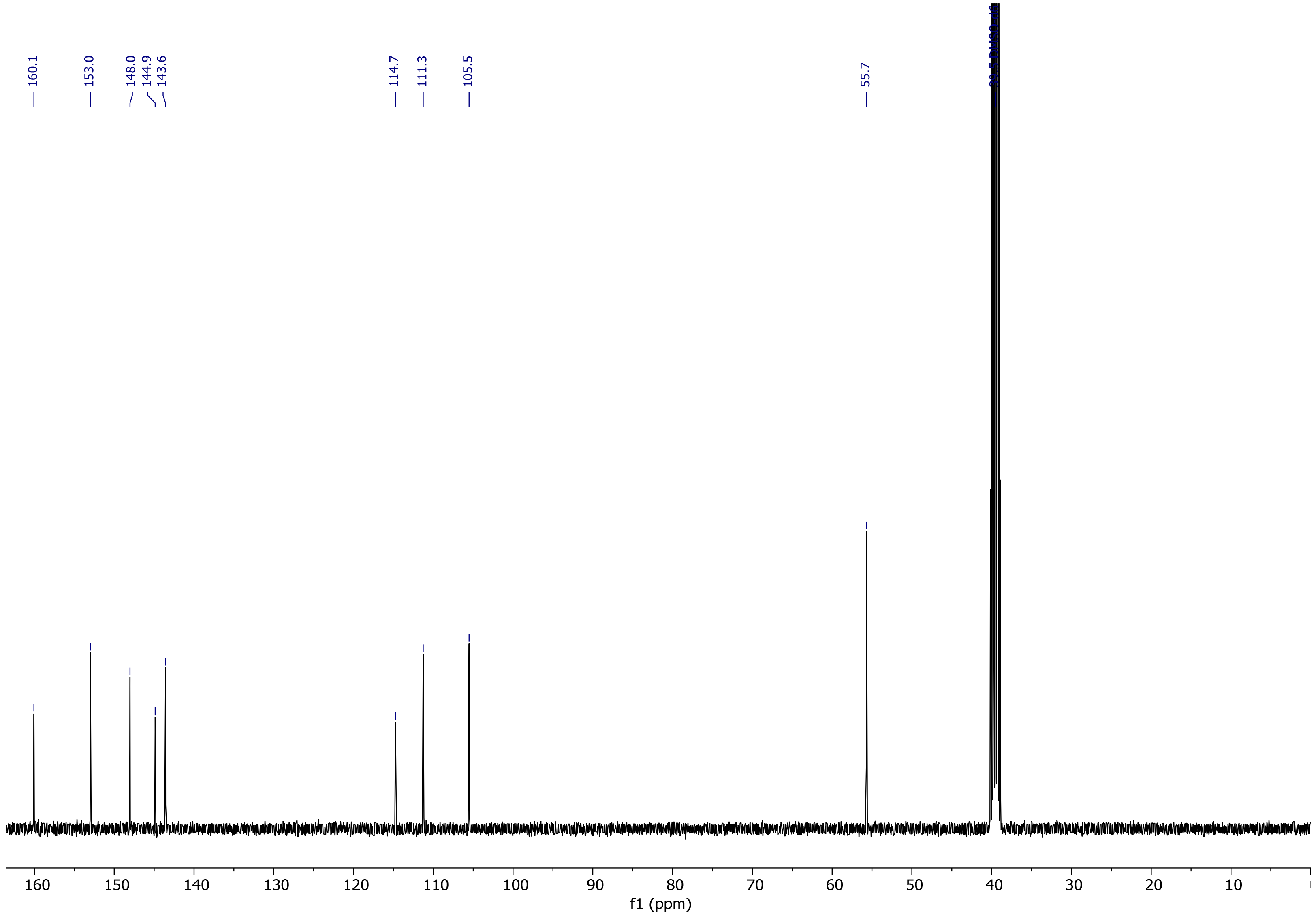

Figure S8. ^{1}H NMR Spectrum (500 MHz) of 7-methoxy-8-hydroxy cycloanthranilylproline (**3**) in CD_3OD

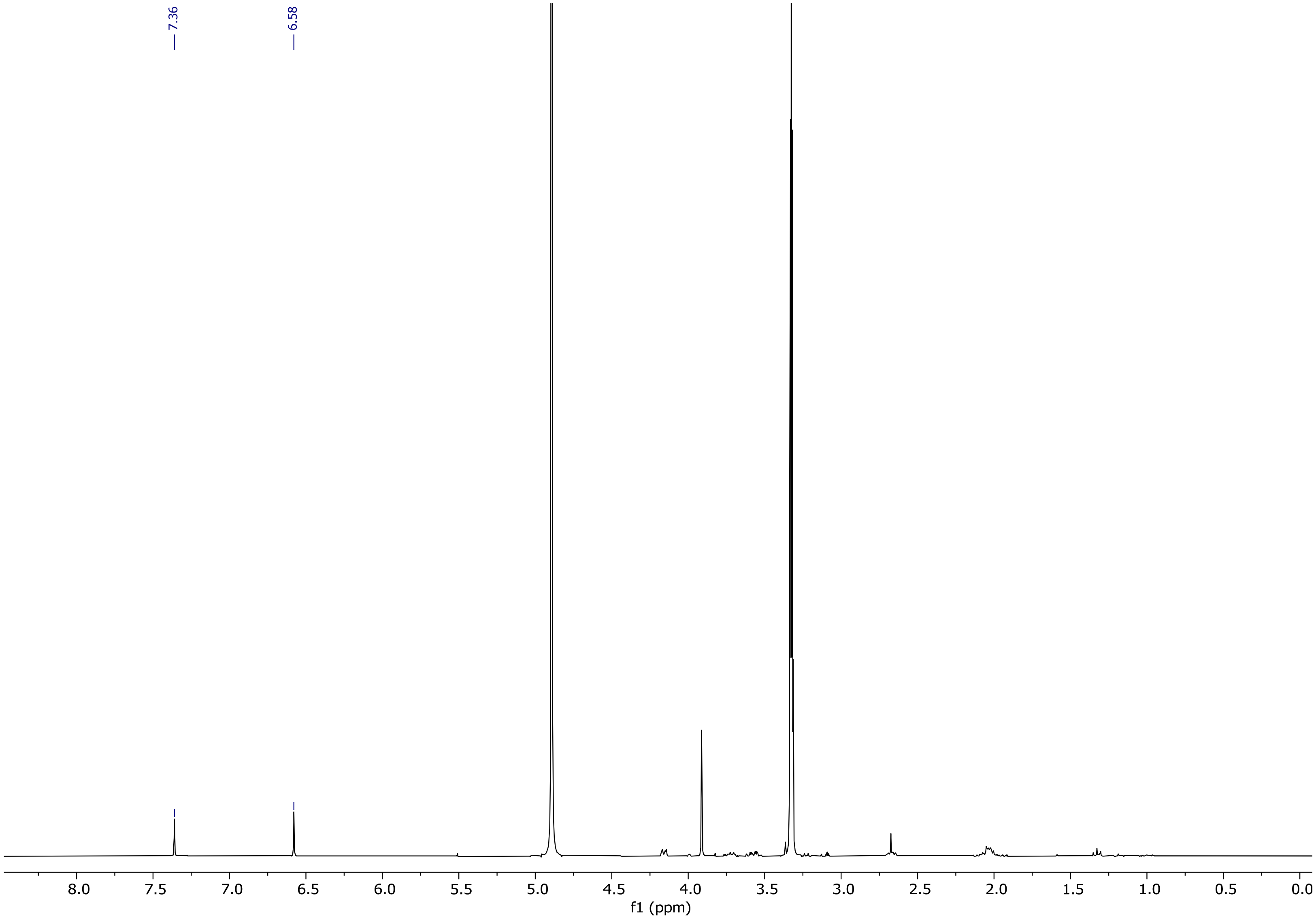

Figure S9. ^{13}C NMR Spectrum (125 MHz) of 7-methoxy-8-hydroxy cycloanthranilylproline (**3**) in CD_3OD

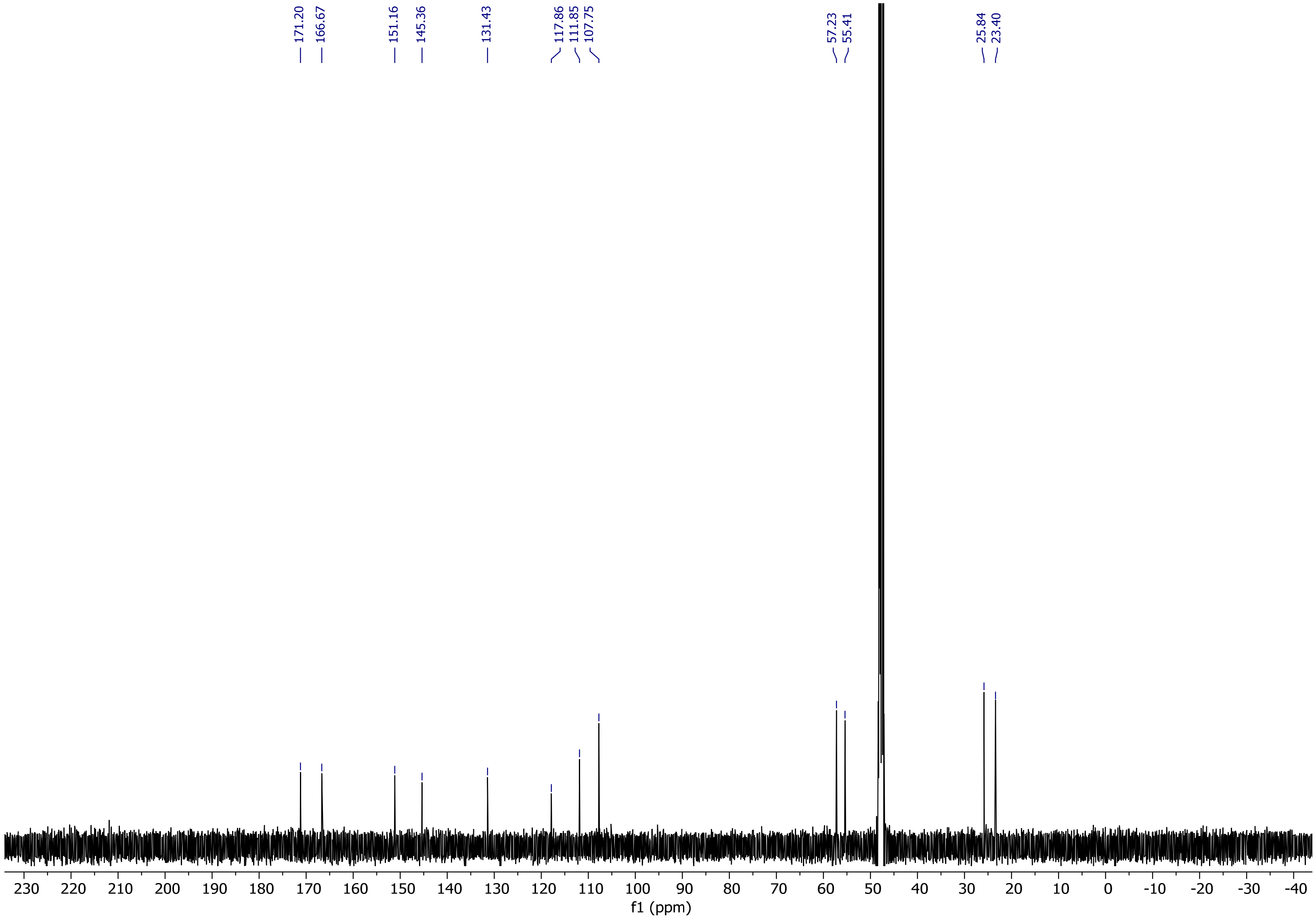

Figure S10. IR Spectrum of actinoquinazolinone (**1**)

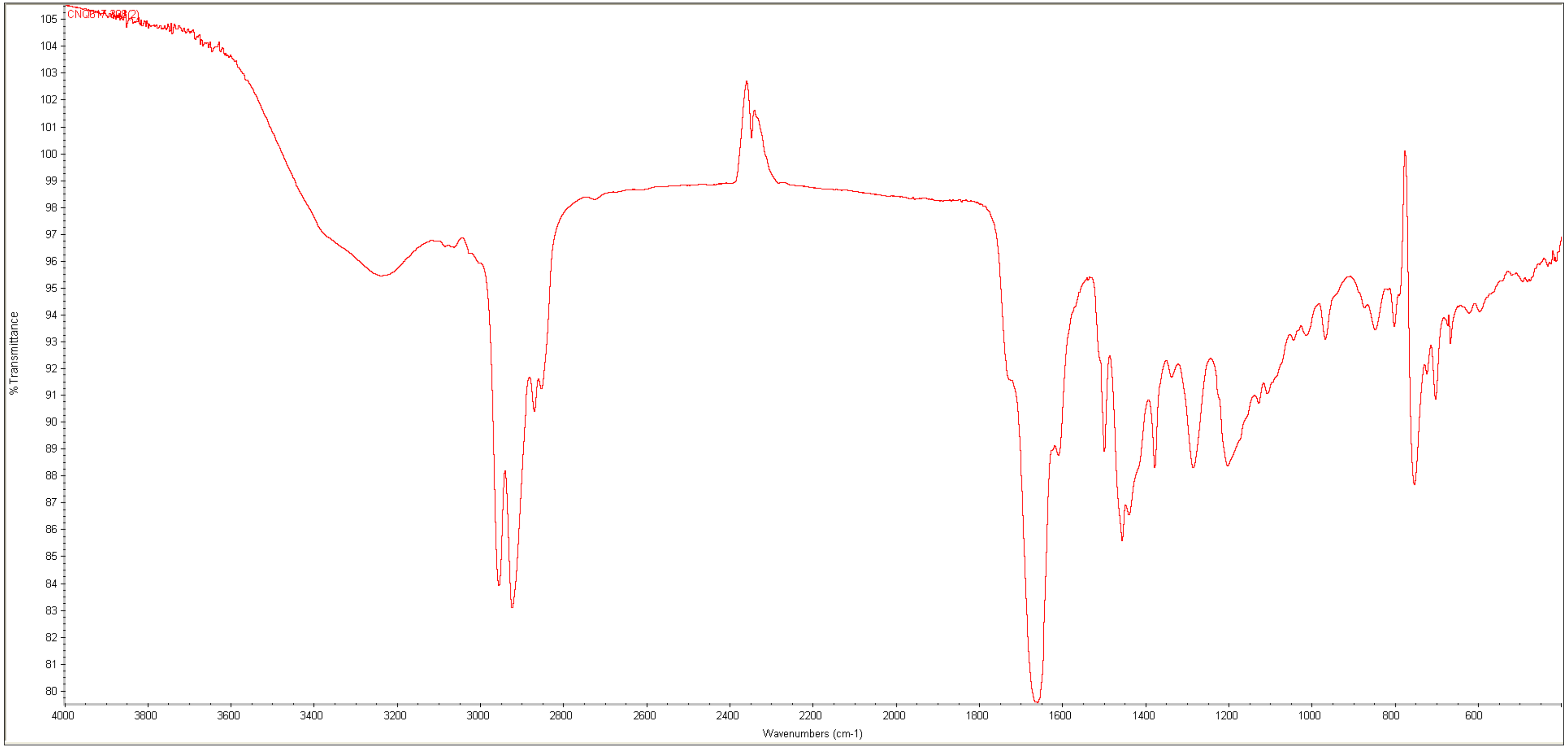

Figure S11. HRMS Spectrum of actinoquinazolinone (**1**)

[Mass Spectrum]
Data : FAB-D178 Date : 17-Feb-2022 13:45
RT : 0.05 min Scan# : (2,4)
Elements : C 100/0, H 100/0, N 5/0, O 10/0
Mass Tolerance : 10ppm, 5mmu if m/z < 500, 10mmu if m/z > 1000
Unsaturation (U.S.) : -0.5 - 20.0

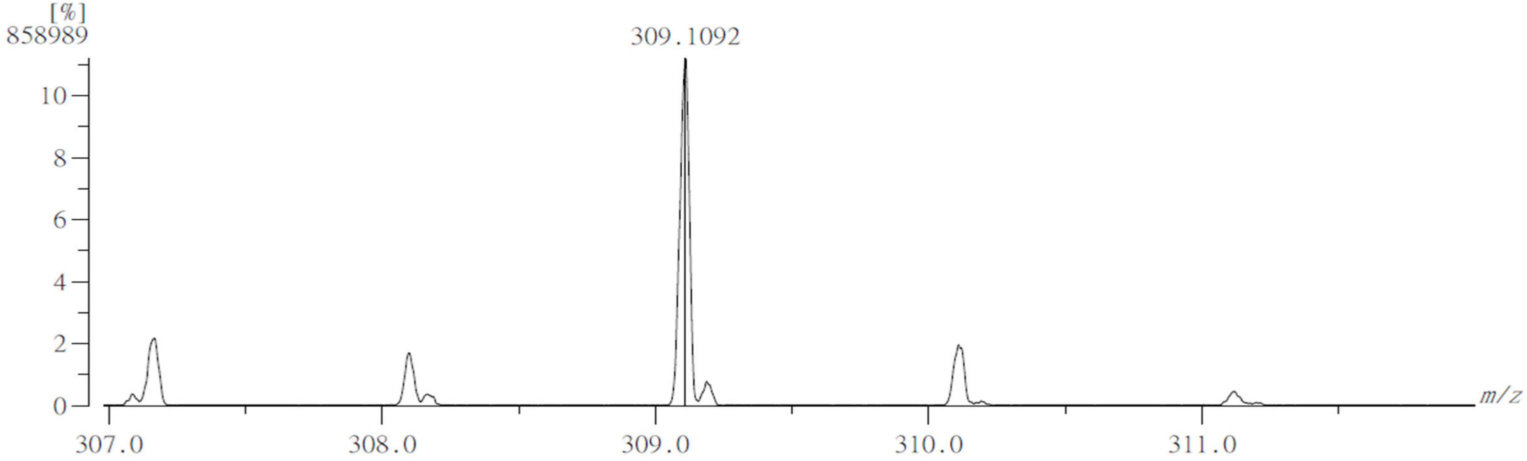

	Observed m/z	Int%	Err[ppm / mmu]	U.S.	Composition
1	309.1092	11.21	-15.6 / -4.8	16.5	C20 H13 N4
2			-6.9 / -2.1	12.0	C17 H15 N3 O3
3			-11.3 / -3.5	11.5	C19 H17 O4
4			+6.1 / +1.9	8.0	C12 H15 N5 O5
5			+1.7 / +0.5	7.5	C14 H17 N2 O6
6			+14.8 / +4.6	3.5	C9 H17 N4 O8
7			+10.4 / +3.2	3.0	C11 H19 N O9
